# Altered lipid metabolites accelerate early dysfunction of T cells in HIV-infected rapid progressors by impairing mitochondrial function

**DOI:** 10.3389/fimmu.2023.1106881

**Published:** 2023-02-17

**Authors:** Si-Yao Li, Lin-Bo Yin, Hai-Bo Ding, Mei Liu, Jun-Nan Lv, Jia-Qi Li, Jing Wang, Tian Tang, Ya-Jing Fu, Yong-Jun Jiang, Zi-Ning Zhang, Hong Shang

**Affiliations:** ^1^ National Health Commission (NHC) Key Laboratory of Acquired Immunodeficiency Syndrome (AIDS) Immunology (China Medical University), National Clinical Research Center for Laboratory Medicine, The First Hospital of China Medical University, Shenyang, China; ^2^ Key Laboratory of AIDS Immunology, Chinese Academy of Medical Sciences, Shenyang, China; ^3^ Units of Medical Laboratory, Chinese Academy of Medical Sciences, Shenyang, China; ^4^ Department of Clinical Laboratory, Cancer Hospital of China Medical University, Liaoning Cancer Hospital & Institute, Liaoning, China

**Keywords:** HIV rapid progression, immunometabolism, metabolomics, lipid metabolites, T-cell dysfunction, mitochondrial ROS

## Abstract

The complex mechanism of immune-system damage in HIV infection is incompletely understood. HIV-infected “rapid progressors” (RPs) have severe damage to the immune system early in HIV infection, which provides a “magnified” opportunity to study the interaction between HIV and the immune system. In this study, forty-four early HIV-infected patients (documented HIV acquisition within the previous 6 months) were enrolled. By study the plasma of 23 RPs (CD4^+^ T-cell count < 350 cells/µl within 1 year of infection) and 21 “normal progressors” (NPs; CD4^+^ T-cell count > 500 cells/μl after 1 year of infection), eleven lipid metabolites were identified that could distinguish most of the RPs from NPs using an unsupervised clustering method. Among them, the long chain fatty acid eicosenoate significantly inhibited the proliferation and secretion of cytokines and induced TIM-3 expression in CD4^+^ and CD8^+^ T cells. Eicosenoate also increased levels of reactive oxygen species (ROS) and decreased oxygen consumption rate (OCR) and mitochondrial mass in T cells, indicating impairment in mitochondrial function. In addition, we found that eicosenoate induced p53 expression in T cells, and inhibition of p53 effectively decreased mitochondrial ROS in T cells. More importantly, treatment of T cells with the mitochondrial-targeting antioxidant mito-TEMPO restored eicosenoate-induced T-cell functional impairment. These data suggest that the lipid metabolite eicosenoate inhibits immune T-cell function by increasing mitochondrial ROS by inducing p53 transcription. Our results provide a new mechanism of metabolite regulation of effector T-cell function and provides a potential therapeutic target for restoring T-cell function during HIV infection.

## 1 Introduction

A proportion of HIV infected individuals progress to acquired immune deficiency syndrome (AIDS) within the first 2–3 years of HIV infection, and are termed “rapid progressors” (RPs). RPs develop severe damage to their immune system early in the infection (1–3). Hence, RPs provide a “magnified” opportunity to: (i) master the mechanisms of rapid progression of immune-system damage; (ii) discover innovative biological markers to predict HIV disease progression; and (iii) identify intervention targets that improve clinical outcomes.

Studies have revealed important mechanisms of the immune response to HIV infection at the genomic, transcriptomic, and proteomic levels (4, 5). The metabolome is a downstream product of transcription and translation and is, thus, at a systems level more directly related to the phenotype (6). A wealth of evidence has emerged that illustrates that the changes in immune cell metabolic pathways can alter their development, fate, and function in the context of physiologic processes, as well as in anti-tumoral and anti-microbial defenses (7–11). HIV infection also leads to deregulated oxidative stress, as well as abnormalities in tryptophan levels, glucose metabolism, and lipid metabolism (12, 13). Studies have reported significant changes in plasma metabolites, including glucose, lipids, and amino acids, at different stages of HIV infection, and these changes are accompanied by alterations in nutrient transport receptors on immune cells (14, 15). However, whether immunometabolism plays a role in HIV disease progression (especially in rapid progression) has not been fully elucidated. Tarancon-Diez, L et al. found that the metabolomic profiles of elite controllers who spontaneously lost virological control were characterized by predominantly aerobic glycolytic metabolism, deregulated mitochondrial metabolism, oxidative stress and increased immunological activation (16). To our best knowledge, there are two studies that have enrolled HIV infected RPs (15, 17). However, due to the small study cohorts (fewer than 10 patients in each group), different factors (e.g., infection duration, treatment), and lack of a functional study, the immunometabolic characteristics of HIV-infected RPs could not be clarified.

T-cell dysfunction established in the early stages of HIV infection represents an almost insurmountable barrier to the immune-mediated control of viral load and the elimination of HIV-infected cells (18). Maintaining a specific metabolic profile is important for T cells to sustain their effector functions. Dysfunctional T cells have been reported to have a metabolic profile characterized by mitochondrial dysfunction, downregulation of genes involved in the TCA cycle, and unbalanced lipid metabolism (19–23). In acute infection, the development of polyfunctional effector CD4+ and CD8+ T cells strongly upregulate mTORC1 and aerobic glycolysis to sustain the energetic demands that are required for the execution of their functions (20). After HIV infection, some patients fail to maintain effective T-cell function and become RPs within 1 year of infection. What are the intrinsic metabolic determinants of this rapid progression in early HIV infection? Identification of the altered metabolites in HIV RPs and investigating their influence on immune T-cell early dysfunction are important to answer the question, which remains largely unknown.

In the present study, we investigated the plasma metabolomics from 44 early HIV-infected (EHI) patients, including 23 RPs and 21 NPs. We first found that rapid disease progression could be predicted by an 11-lipid metabolite signature. Then, we studied the role of these metabolites on T-cell function. Our in vitro study showed that one of the lipid metabolites, eicosenoate, inhibited T-cell immune function in HIV infection by increasing mitochondrial ROS via p53 transcription induction. Finally, we found that the mitochondrial-targeting antioxidant mito-TEMPO restored eicosenoate-induced inhibition of T-cell function. Our results provide new insights into the pathogenesis of HIV infection.

## 2 Materials and methods

### 2.1 Study participants

Based on a large-scale, open, prospective cohort set up in our HIV Voluntary Counseling and Testing Center, we recruited 44 treatment-naive EHI patients with different disease progression. All EHI patients were defined by documented HIV acquisition within the previous 6 months (from 2008 to 2013) and were followed up. Patients were divided into two groups; 23 patients were RPs (CD4+ T cell < 350 cells/μl within 1 year of infection) and the remaining 21 HIV infected patients were NPs (CD4+ T cell > 500 cells/μl after 1 year of infection). **Table 1** summarizes relevant characteristics of the study groups. Peripheral blood samples were collected after an overnight fast. Plasma was obtained and stored at −80°C until analyses. Metabolite profiles in plasma were detected at ~120 days of HIV infection. Twenty age-, sex-, and lifestyle-matched healthy persons were enrolled as HIV-negative controls (NCs). For the in vitro experiments, HIV-infected individuals from our AIDS clinic were included. Ethical approval of the study protocol was obtained from the local Ethics Review Committee. Written informed consents were obtained from all individuals participating in this study.

**Table 1:** Clinical characteristics of patients with early HIV infection and HIV negative controls.

Characteristic	RPs	NPs	NCs
N	23	21	20
Age, mean (SD), years	29 (8)	25 (6)	26 (4)
Male (number, %)	20 (100)	23 (100)	21 (100)
CD4 (cells/µL), mean (SD)	316 (99)	683 (152)	747 (198)
Subtype CRF01AE (No, %)	19 (83)	14 (67)	
Viral load (log copies/mL), mean (SD)	4.54 (0.9)	3.72 (0.98)	
Estimated days of infection, mean (SD)	112 (31)	107 (23)	

RPs, rapid progressors; NPs, normal progressors; NCs, HIV negative controls.

### 2.2 Metabolomic profiling and bioinformatics analysis

Metabolomic profiling was performed by Liquid chromatography-tandem mass spectrometry (LC-MS/MS) coupled to gas chromatography-mass spectroscopy (GC-MS). In brief, a recovery standard was added before the first step in the extraction process for quality control (QC) purposes. To remove proteins, dissociated small molecules bound to proteins or trapped in the precipitated protein matrix. Proteins were precipitated with methanol to recover chemically diverse metabolite. The resulting extract was divided into five fractions, one each for: (i) analyses by ultra-high pressure liquid chromatography-tandem mass spectrometry (UPLC-MS/MS) with positive-ion mode electrospray ionization; (ii) analyses by UPLC-MS/MS with negative-ion mode electrospray ionization; (iii) LC polar platform; (iv) analyses by GC-MS; (v) backup. Samples were placed briefly on a TurboVap® evaporator (Zymark, Hopkinton, MA, USA) to remove the organic solvent. For LC, samples were stored overnight under nitrogen before preparation for analyses. For GC, each sample was dried under vacuum overnight before preparation for analyses. Instrument variability was determined by calculating the median relative standard deviation (RSD) for the internal standards that were added to each sample before injection into the mass spectrometer. The variability of the overall process was determined by calculating the median RSD for all endogenous metabolites (i.e., non-instrument standards) present in 100% of the matrix samples, which were technical replicates of pooled client samples.

Raw data were extracted, and peaks were identified and processed for QC using Metabolon hardware and software (Morrisville, NC, USA). For studies spanning multiple days, a data-normalization step was undertaken to correct the variation resulting from inter-day tuning differences in the instrument. Each compound was corrected in run-day blocks by registering the median values to equal 1.00 and normalizing each data point proportionately. For a single-day run, this is equivalent to the raw data. Each biochemical in OrigScale is rescaled to set the median equal to 1.00 and expressed as imputed normalized counts for each biochemical (Scaled ImpData). Data were normalized to correct for the variation resulting from differences in inter-day tuning of the instrument. Raw area counts for a compound were divided by the median value, setting the median values equal for the run on each day (24, 25). Missing values were imputed with its observed minimum after the normalization step for each metabolite (26). After normalization and imputation, the data were log-transformed. Metabolomic detection and analysis was performed by the Shanghai Jiao Tong University–Metabolon Joint Metabolomics Laboratory (Shanghai, China).

### 2.3 Culture and stimulation of cells

Peripheral blood mononuclear cells (PBMCs) from HIV infected patients were obtained by Ficoll–Hypaque density gradient centrifugation. Primary CD3+ T cells were isolated from PBMCs by selection with magnetic beads using a CD3+ T-cell isolation kit (cell purity > 95%; Stem Cell Technologies, Vancouver, Canada). Freshly isolated T cells were cultured in round-bottomed 96-well culture plates (Corning, NY, USA) at a final density of 2.5×10^6^ cells/mL in RPMI 1640 media (HyClone, Logan, UT, USA) supplemented with 10% fetal bovine serum. The 96-well culture plates were incubated at 37°C in an incubator in an atmosphere of 5% CO2.

To determine the responses of T cells to fatty acid stimulation, T cells were stimulated with anti-CD3/CD28 coated Dynabeads (4:1 ratio) (ThermoFisher Scientific, Waltham, MA, USA) and 500μM eicosenoate for 24 h (Cayman Chemicals, Ann Arbor, MI, USA). Dimethyl sulfoxide (DMSO) was used as the control. In order to evaluate the effects of p53 on fatty acid eicosenoate-treated T-cell mitochondrial function, primary isolated T cells were co-treated with the p53 inhibitor pifithrin-α (20μM; Santa Cruz Biotechnologies, Santa Cruz, CA, USA) for 24 h (27, 28). To demonstrate the effect of mitochondrial reactive oxygen species (ROS) on eicosenoate treated T-cell function, mito-TEMPO (200μM, Sigma-Aldrich, MO, USA), a mitochondrial-targeting antioxidant, was co-treated with T cells for 24 h.

### 2.4 Staining and flow cytometric analysis

To explore the effect of fatty acids on T-cell exhaustion, isolated T cells were stained with APC-Cy7-conjugated anti-CD4, PerCP-Cy5.5-conjugated anti-CD8, PE-conjugated anti-TIM3, or BV421-conjugated anti-TIM3 (Biolegend, San Diego, CA, USA) for 30 mins at 4°C. To investigate the effects of fatty acids on T-cell proliferation, isolated T cells were marked with Cell Trace™ Violet (Thermo Fisher Scientific, Waltham, MA, USA) for 20 mins at 37°C, conforming to manufacturer’s instructions, washed with complete medium, and cultured in the presence of anti-CD3/CD28-coated Dynabeads. After incubation for 5 d, dead cells were excluded by staining with 7-aminoactinomycin D. To investigate the effects of fatty acids on CD4+ and CD8+ T-cell function, isolated T cells were activated with anti-CD3/CD28-coated Dynabeads and pre-incubated with PE-conjugated anti-CD107a for 24 h. The protein-transport inhibitor (GolgiStop; 1 μl/mL, BD Biosciences) was added to the culture for the final 6 h. After that, cells were stained with LIVE/DEAD fixable dead cell stain reagent (Invitrogen, Carlsbad, CA, USA), APC-Cy7-conjugated anti-CD4 and PerCP-Cy5.5-conjugated anti-CD8 (Biolegend, San Diego, CA, USA). Subsequently, for intracellular staining, cells were incubated with Fixation/Permeabilization working solution (eBioscience, CA, USA) for 30 mins in the dark, followed by incubation with BV421-conjugated anti-interferon (IFN)-γ and APC-conjugated anti-IL-2 for 30 mins at 4°C. To investigate the effects of fatty acid on CD4+ and CD8+ T cell activation, isolated T cells were activated with anti-CD3/CD28-coated Dynabeads and stained with Percp-Cy5.5-conjugated anti-CD8, APC-Cy7-conjugated anti-CD4, BV786-conjugated anti-CD69, BV421-conjugated anti-CD25 and BV510-conjugated anti-HLA-DR (Biolegend, San Diego, CA, USA). To explore the effects of fatty acids on mitochondrial mass and ROS, T cells co-incubated with fatty acids for 24 h were resuspended in warmed 37°C staining solution containing MitoTracker® Green FM (50nM; Thermo Fisher Scientific) and MitoSOX™ Red Mitochondrial Superoxide Indicator (5μM; Thermo Fisher Scientific) for 30 mins, washed the cells with PBS, and then stained with the LIVE/DEAD™ Fixable Aqua Dead Cell Stain kit. To investigate the effect of mito-TEMPO on T cell exhaustion, function and mitochondrial ROS, T cells were co-incubated with 200μM mito-TEMPO and 500μM eicosenoate and pre-incubated with PE-conjugated anti-CD107a for 24 h. Then the cells were stained with MitoSOX™ Red Mitochondrial Superoxide Indicator, PE-conjugated anti-TIM3, BV421-conjugated anti-PD-1 and BV421-conjugated anti-interferon (IFN)-γ. Cells were examined using a flow cytometer (BD LSR II; BD Biosciences, San Jose, CA, USA), and data were analyzed using FlowJo software (Ashland, OR, USA).

### 2.5 Seahorse extracellular flux analysis

On the day prior to the assay, the Agilent Seahorse XFp Sensor Cartridge with XF Calibrant was hydrated in a non-CO2 37°C incubator overnight. Isolated T cells co-treated with eicosenoate were stimulated with ImmunoCult Human CD3/CD28 T-cell Activator (StemCell Technologies, Vancouver, Canada) for 24 h. On the day of the assay, cells were resuspended in warmed assay medium to the desired concentration (5×10^5^ cells in 50μl/well) before seeding them onto the CellTak-coated Seahorse Cell Culture Miniplate (wells A and H were used as background correction wells). Then, cells were centrifuged at 350×g for 5 mins, and 130μl assay medium was added to each well for a final volume of 180μl. Finally, the Miniplate was transferred to a non-CO2 37°C incubator for 20 mins to ensure that the cells were entirely stable. Oxygen consumption rate (OCR) was measured without adding any drug (basal respiration), followed by measurement of OCR changes upon subsequent addition of 1.5 μM ATP synthase inhibitor oligomycin and 1 μM carbonyl cyanide-4 (trifluoromethoxy), and phenylhydrazone (FCCP). Finally, 0.5 μM rotenone and 0.5 μM antimycin A were injected to completely inhibit mitochondrial respiration by blocking complex I and complex III. Basal respiration, maximal respiration, and spare respiration were analyzed using an XFp Cell Mito Stress Tests Kit on an Agilent Seahorse XF HS Mini instrument, according to the corresponding operation protocol.

### 2.6 Analysis of microarray data

To explore the underlying mechanism of impaired cell and mitochondria function with fatty acid eicosenoate, microarray data from Gene Expression Omnibus (GEO) with accession number GSE44216 we downloaded. Using the online GEO2R analysis tool, differentially expression genes (DEGs) between RPs and NPs with adjust p value <0.05 and fold change (FC) >1.5 were identified. Kyoto Encyclopedia of Genes and Genomes (KEGG) pathway enrichment was analyzed on the DAVID website.

### 2.7 Reverse transcription and quantitative real-time polymerase chain reaction

mRNA was extracted from T cells using the mRNeasy™ Micro kit (Qiagen, Stanford, VA, USA). RNA was reverse transcribed using the Primpscript® RT reagent kit (TaKaRa Biotechnology, Shiga, Japan) according to the corresponding instructions. qRT-PCR for mRNA detection was carried out using SYBR® Premix Ex Taq™ II (TaKaRa Biotechnology). The p53 mRNA expression was normalized to the glyceraldehyde 3-phosphate dehydrogenase (GAPDH) mRNA expression level. The following sequences of primers were used: for RT-PCR of p53: 5’ CCACCATCCACTACAACTACAT 3’ (forward);5’ AAACACGCACCTCAAAGC 3’ (reverse); For GAPDH: 5’ GAAGGTGAAGGTCGGAGTC 3’ (forward); 5’ GAAGATGGTGATGGGATTTC 3’ (reverse). Relative expression of mRNA was calculated based on the change in the number of cycles using the 2−ΔΔCt method.

### 2.8 Statistical analyses

Principal component analysis (PCA) was performed using Origin 9.1 (OriginLab, Northampton, MA, USA) to analyze the distribution of metabolites in NCs and EHIs. Welch’s two-sample t-test was used to identify plasma metabolites that differed significantly between different groups of individuals. For analyses of receiver operating characteristic (ROC) curves of the 11-metabolites signature combination, P (probability of a patient sample) was calculated for inclusion in ROC analyses using the formula:


$$\text{X }=\text{ logit }\left(\text{P}\right)\;=\text{ ln }(\text{P}/1-\text{P})\;=\;{\text{b}}_{0}+{\text{b}}_{1}{\text{E}}_{1}+{\text{b}}_{2}{\text{E}}_{2}+{\text{b}}_{3}{\text{E}}_{3}+\ldots^{+}{\text{b}}_{\text{n}}\text{Δ}{\text{E}}_{\text{n}}$$

where b was the regression coefficient by binary logistic regression, E was the expression of each metabolite, X = −3.378 − 1.097 × 3-hydroxydecanoate + 1.581 × eicosenoate − 0.283 × 9,10-DiHOME + 0.411 × 10-nonadecenoate + 1.109 × 12,13-DiHOME + 2.649 × caprate + 0.835 × caprylate − 0.836 × myristoleate + 1.66 × tetradecanedioate − 0.474 × cholate − 1.154 × deoxycholate, and P = eX/(1 + eX). The Youden Index was used as a criterion for selecting the optimum cut-off point. HIV-infected individuals were divided into two groups according to the Youden Index (high or low). Kaplan−Meier techniques were utilized to determine the effect of metabolite expression on time-dependent disease progression (CD4+ T cell < 350 cells/μl was considered as the endpoint for follow-up).

For the in vitro experiments, parametric t-test or paired t-test was used if the data was Gaussian. If the data was not Gaussian, nonparametric Mann-Whitney test or Wilcoxon matched-pairs signed rank test was used. For the comparisons of data sets, one-way ANOVA was used to compare the data with normal distribution and Friedman test was employed for data sets not with normal distribution. Data analyses were performed using Prism 5.0 (GraphPad, San Diego, CA, USA). P < 0.05 was considered significant.

## 3 Results

### 3.1 The profile and signature of metabolites can be used to differentiate between RPs and NPs

**Figure 1:**
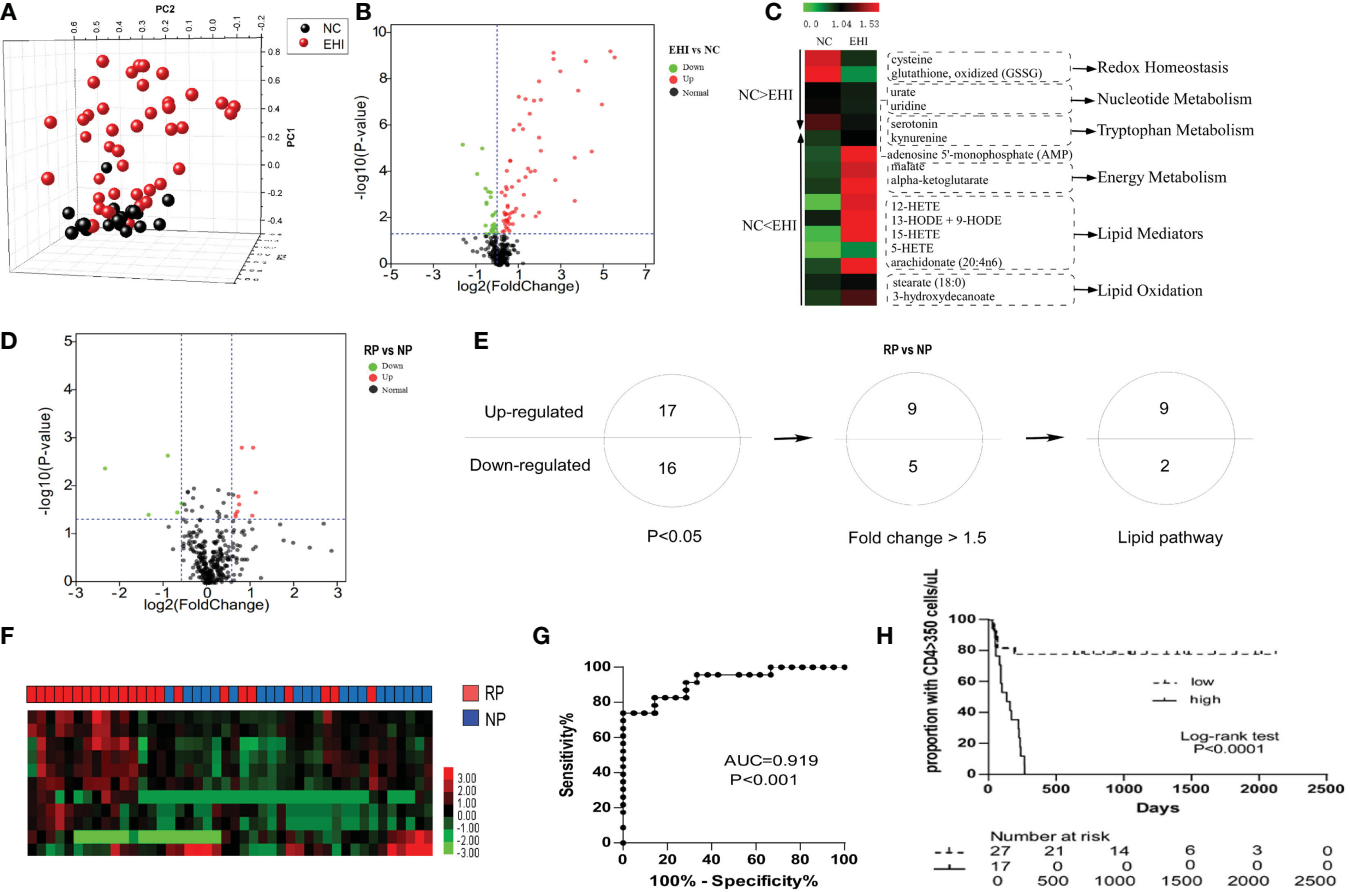
The metabolomic profile of HIV-infected patients. **(A)** Principal component analysis (PCA; Origin 9.1) plot of 352 plasma metabolites from patients with early HIV infection (EHI, n = 44) at ~120 days of infection and HIV-negative controls (NCs, n = 20). The metabolomic profile of the EHI group represented by these metabolites was distinct from that of NCs according to PCA. **(B)** Volcano plot of 352 plasma metabolites from EHIs and NCs. Ninety-one metabolites were significantly different between EHIs and NCs (*P* < 0.05). **(C)** Pathways associated with redox homeostasis, nucleotide metabolism, tryptophan metabolism, energy metabolism, lipid mediators, and lipid oxidation, based on analyses of metabolites. **(D)** Volcano plot of 352 plasma metabolites from RPs and NPs. Thirty-three metabolites were significantly different between rapid progressors (RPs) and normal progressors (NPs; *P* < 0.05). **(E)** Thirty-three differentially expressed metabolites in RPs and NPs (*P* < 0.05), including 14 metabolites with a fold-change > 1.5, were noted. Among them, 11 were lipid metabolites. **(F)** Heatmap demonstrating the 11-lipid metabolites that were differentially expressed between RPs and NPs by unsupervised hierarchical clustering. **(G)** Analyses of ROC curves revealed that the 11-lipid metabolite signature had a predictive accuracy of 91.9%, as measured by AUC, for rapid disease progression. **(H)** According to the Youden Index (high or low), high expression of the 11-lipid metabolite signature was highly predictive of disease progression.

The early immune response to HIV infection is likely to be an important factor in determining the clinical course of the disease (29). First, we studied the plasma metabolomes of 44 patients with EHI at approximately 120 days of infection and compared them with those of NCs. Three hundred and fifty-two metabolites were identified in this study. The metabolomic profile of the EHI group represented by these metabolites was distinct from that of NCs according to PCA (**Figure 1A**). Of the 352 compounds examined, 91 were differentially expressed between EHIs and NCs (**Figure 1B**), including 65 upregulated and 26 downregulated compounds in EHIs (**Supplemental Table 1**). Among them, the metabolites associated with redox homeostasis, nucleotide metabolism, tryptophan metabolism, energy metabolism, lipid mediators, and lipid oxidation were identified (P < 0.05) (**Figure 1C**). The results show that deregulated immunometabolism occurs early in chronic HIV infection (12).

Next, we sought out biomarkers which could differentiate disease progression in early HIV infection. By studying the metabolomics differences between RPs and NPs, 33 differentially expressed metabolites were identified between RPs and NPs (P < 0.05) (**Figure 1D**; **Supplemental Table 2**). Among them, 17 metabolites were upregulated and 16 were downregulated (P < 0.05) in RPs. Among these 33 compounds, there were 14 metabolites significantly differentially expressed between RPs and NPs with a fold-change > 1.5. Eleven of the significantly differentially expressed metabolites were lipid metabolites (**Figure 1E**; **Supplemental Table 2**). We, therefore, mined our data for these lipid metabolites to ascertain if they had an impact on HIV disease progression. The predictive power of the 11-substance signature for HIV disease progression was determined. With complete linkage used in the unsupervised clustering method, most of the RPs could be distinguished from NPs using these 11 lipid metabolites (**Figure 1F**). According to ROC analyses, the 11 lipid metabolites signature had a predictive accuracy of 91.9% for rapid disease progression (P < 0.001) (**Figure 1G**). Patients were divided into two groups according to the Youden Index (high or low), and the 11-lipid metabolites signature was highly predictive of disease progression in the high index group (P < 0.0001) (**Figure 1H**).

### 3.2 The lipid metabolite eicosenoate impairs T-cell function

**Figure 2:**
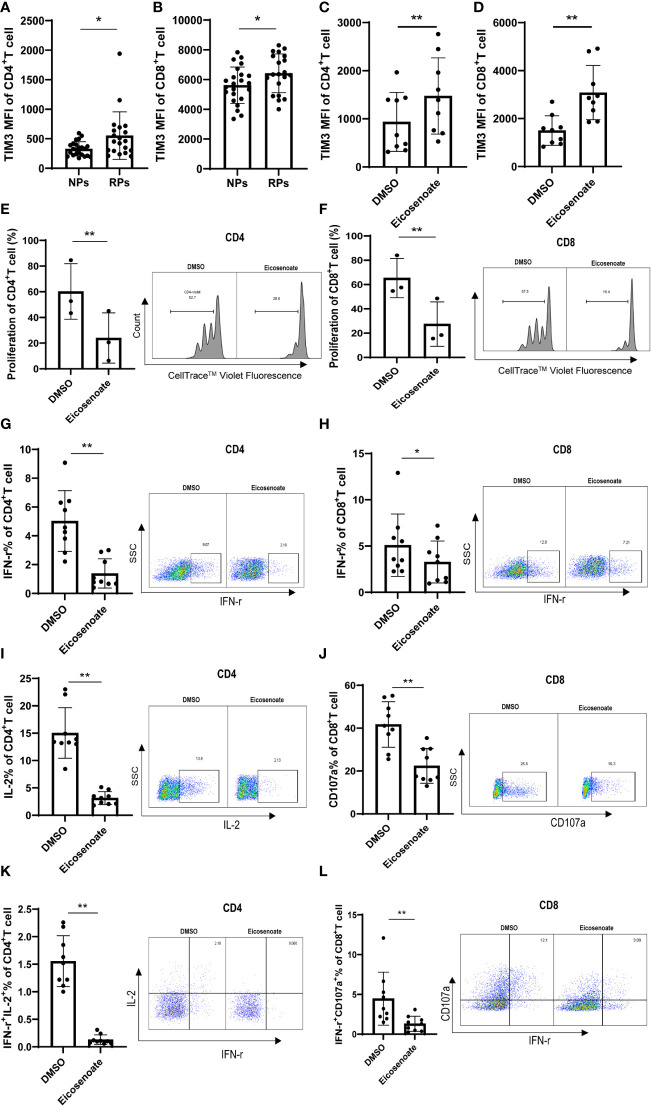
The lipid metabolite eicosenoate impairs T-cell function. To investigate the responses of cells to lipid metabolites, CD3^+^ T cells were stimulated with anti-CD3/CD28-coated Dynabeads (4:1 ratio) and 500μM of the lipid metabolite eicosenoate simultaneously. **(A, B)** Exhaustion indicator TIM3 mean fluorescence intensity (MFI) on CD4^+^ (*P* < 0.05) and CD8^+^ (*P* < 0.05) T cells in rapid progressors (RPs; n = 20) was higher than that in normal progressors (NPs; n = 23). **(C, D)** CD3^+^ T cells were co-incubated with DMSO or eicosenoate plus anti-CD3/CD28-coated Dynabeads (4:1 ratio) for 24 h and the expression of TIM3 was detected (n = 9). The expression of TIM3 on the surface of CD4^+^ (*P* < 0.01) and CD8^+^ (*P* < 0.01) T cells was significantly increased after a 24 h incubation with 500μM eicosenoate. **(E, F)** Primary CD3^+^ T cells were labeled with Cell Trace™ Violet and stimuli were added (anti-CD3/CD28-coated Dynabeads and 500μM eicosenoate). After incubation for 5 d, dividing CD4^+^ and CD8^+^ T cells were analyzed. Eicosenoate significantly inhibited anti-CD3/CD28-coated Dynabeads-induced proliferation of CD4^+^ (*P* < 0.01) and CD8^+^ T cells (*P* < 0.01) at 500μM (n = 3). **(G-L)** Primary CD3^+^ T cells were stimulated with anti-CD3/CD28-coated Dynabeads (4:1 ratio) and 500 µM eicosenoate for 24 h, and GolgiStop (1 μl/mL) was added to the culture for the final 6 h. IFN-γ expression in anti-CD3/CD28-coated Dynabeads-stimulated CD4^+^ T cells (G, n = 9) (*P* < 0.01) and CD8^+^ T cells (H, n = 9) was significantly reduced (*P* < 0.05) in the presence of 500µM eicosenoate. IL-2 expression in anti-CD3/CD28-coated Dynabeads-stimulated CD4^+^ T cells (I, n = 9) was significantly reduced in the presence of 500µM eicosenoate (*P* < 0.01). CD107a expression in anti-CD3/CD28-coated Dynabeads-stimulated CD8^+^ T cells (J, n = 9) was significantly reduced in the presence of 500µM eicosenoate (*P* < 0.01). IFN-γ and IL-2 co-expression in anti-CD3/CD28-coated Dynabeads-stimulated CD4^+^ T cells (K, n = 9) was significantly reduced in the presence of 500µM eicosenoate (*P* < 0.01). IFN-γ and CD107a co-expression in anti-CD3/CD28-coated Dynabeads-stimulated CD8^+^ T cells (L, n = 9) was significantly reduced in the presence of 500µM eicosenoate (*P* < 0.01). In each panel, representative flow cytograms and comparisons of the parameters between eicosenoate-stimulated T cells and controls are shown. *P < 0.05 and **P < 0.01.* P* values were calculated by Mann-Whitney test **(A, B)**, Wilcoxon matched-pairs signed rank test **(C, D, G-L)**, Paired t test **(E, F)**.

T-cell dysfunction is established in the early stages of HIV infection. The exhaustion marker TIM3 was significantly increased on CD4+ and CD8+ T cells in RPs compared with NPs (**Figures 2A, B**), indicating early dysfunction of T cells. Because lipid metabolites have been linked to impaired T-cell function (30, 31), we hypothesized that altered lipid metabolites have a role in T-cell biology. Among the 11 differentially expressed lipid metabolites, eicosenoate, which was upregulated in RPs, increased TIM3 expression on CD4+ and CD8+ T cells at a concentration of 500μM in vitro (**Figures 2C, D**). In addition, 500μM eicosenoate significantly inhibited the proliferation of CD4+ and CD8+ T cells after stimulation of anti-CD3/CD28 for 5 days (**Figures 2E, F**). Next, we studied the effect of eicosenoate on IFN-γ, IL-2, and CD107a expression in CD3+ T cells. IFN-γ expression in anti-CD3/CD28-induced CD4+ and CD8+ T cells was reduced significantly in the presence of 500μM eicosenoate (**Figures 2G, H, K, L**). IL-2 expression in anti-CD3/CD28-induced CD4+ T cells was diminished after co-incubation with 500μM eicosenoate for 24 h (**Figure 2I, K**). CD107a expression in anti-CD3/CD28-induced CD8+ T cells was decreased after eicosenoate treatment for 24 h (**Figures 2J, L**). Eicosenoate treatment could also decrease the markers of early and later activation, including CD69, CD25, HLA-DR expression (**Supplemental Figure 1**). These data suggest that increased levels of the lipid metabolite eicosenoate in HIV infected RPs plays a role in inhibiting T-cell functions.

### 3.3 The lipid metabolite eicosenoate impairs T-cell mitochondrial function

**Figure 3:**
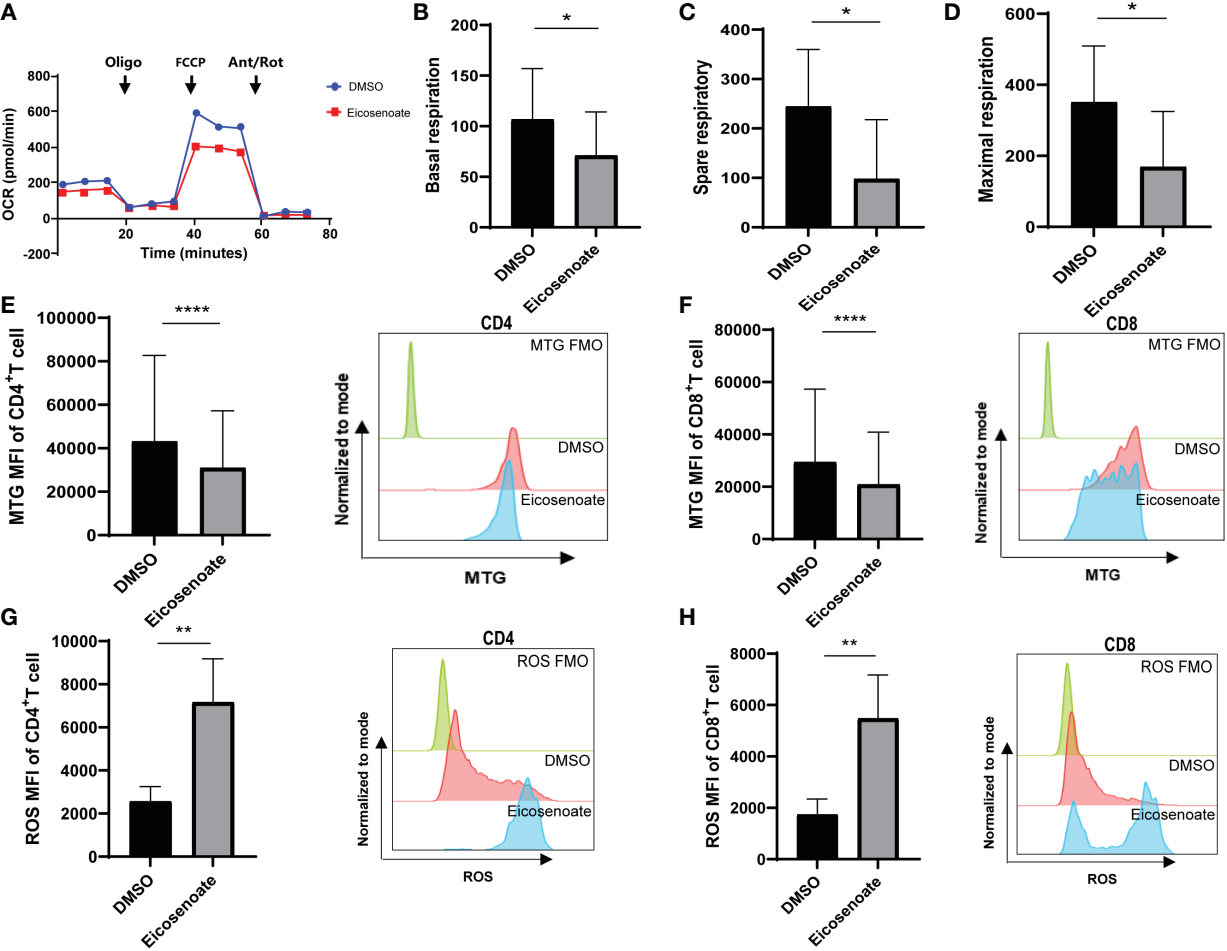
The lipid metabolite eicosenoate impairs T-cell mitochondrial function. **(A)** Oxygen consumption rate (OCR) at baseline and after administration of 1.5μM oligomycin (oligo), 1μM FCCP, and 0.5μM antimycin + rotenone (Ant/Rot) after 24 h stimulation of CD3^+^ T cells with 500μM eicosenoate and ImmunoCult Human CD3/CD28 T-cell Activator. **(B-D)** Basal respiration **(B)**, spare respiratory **(C)** and maximal respiration **(D)** were calculated and compared between eicosenoate-stimulated cells and controls and results are shown (n = 4). **(E, F)** The mitochondrial mass of primary CD3^+^ T cells was marked using MitoTracker Green after co-incubation with 500μM eicosenoate and anti-CD3/CD28-coated Dynabeads (4:1 ratio) for 24 h. Both CD4^+^ (*P* < 0.0001) and CD8^+^ (*P* < 0.0001) T-cell mitochondrial mass were significantly decreased (n = 15). **(G, H)** Mitochondrial ROS in primary CD3^+^ T cells were marked using MitoSOX Red Mitochondrial Superoxide Indicator. After co-incubation with 500μM eicosenoate and anti-CD3/CD28-coated Dynabeads (4:1 ratio) for 24 h, mitochondrial ROS in CD4^+^ (*P* < 0.01) and CD8^+^ (*P* < 0.01) T cells were significantly increased (n = 10). In each panel, representative flow cytograms and comparisons of the parameters between eicosenoate-stimulated cells and controls are shown. *P < 0.05, **P < 0.01, and ****P < 0.0001.* P* values were calculated by Paired t-test **(B-D)**, Wilcoxon matched-pairs signed rank test **(E–H)**.

Recent evidence supports the notion that mitochondrial metabolism is necessary for T-cell activation, proliferation, and function (32). Studies show that long chain fatty acids contribute to CD8+ T-cell dysfunction by damaging mitochondrial function in tumors (22, 33). Whether altered plasma lipid metabolites in HIV RPs influence T-cell mitochondrial function remains unclear. We therefore measured mitochondrial respiratory function in CD3+ T cells after co-incubation with the lipid metabolite eicosenoate (500μM) and CD3/CD28 T-cell activator for 24 h using an OCR assay. Basal respiration, spare respiration, and maximal respiration in CD3+ T-cells were significantly reduced after co-incubation with eicosenoate for 24 h (**Figures 3A–D**). Meanwhile, we performed mitochondrial ROS and mitochondrial mass assays with eicosenoate-treated CD4+ and CD8+ T cells for 24 h. Eicosenoate treatment significantly decreased mitochondrial mass (**Figures 3E, F**) and increased mitochondrial ROS (**Figures 3G, H**). Because reduced mitochondrial respiration capacity and increased mitochondrial ROS indicate that mitochondria are in a state of oxidative stress (34, 35), these data suggest that the lipid metabolite eicosenoate impairs T-cell mitochondrial function.

### 3.4 The p53 pathway participates in eicosenoate-induced impairment of T-cell mitochondrion function

**Figure 4:**
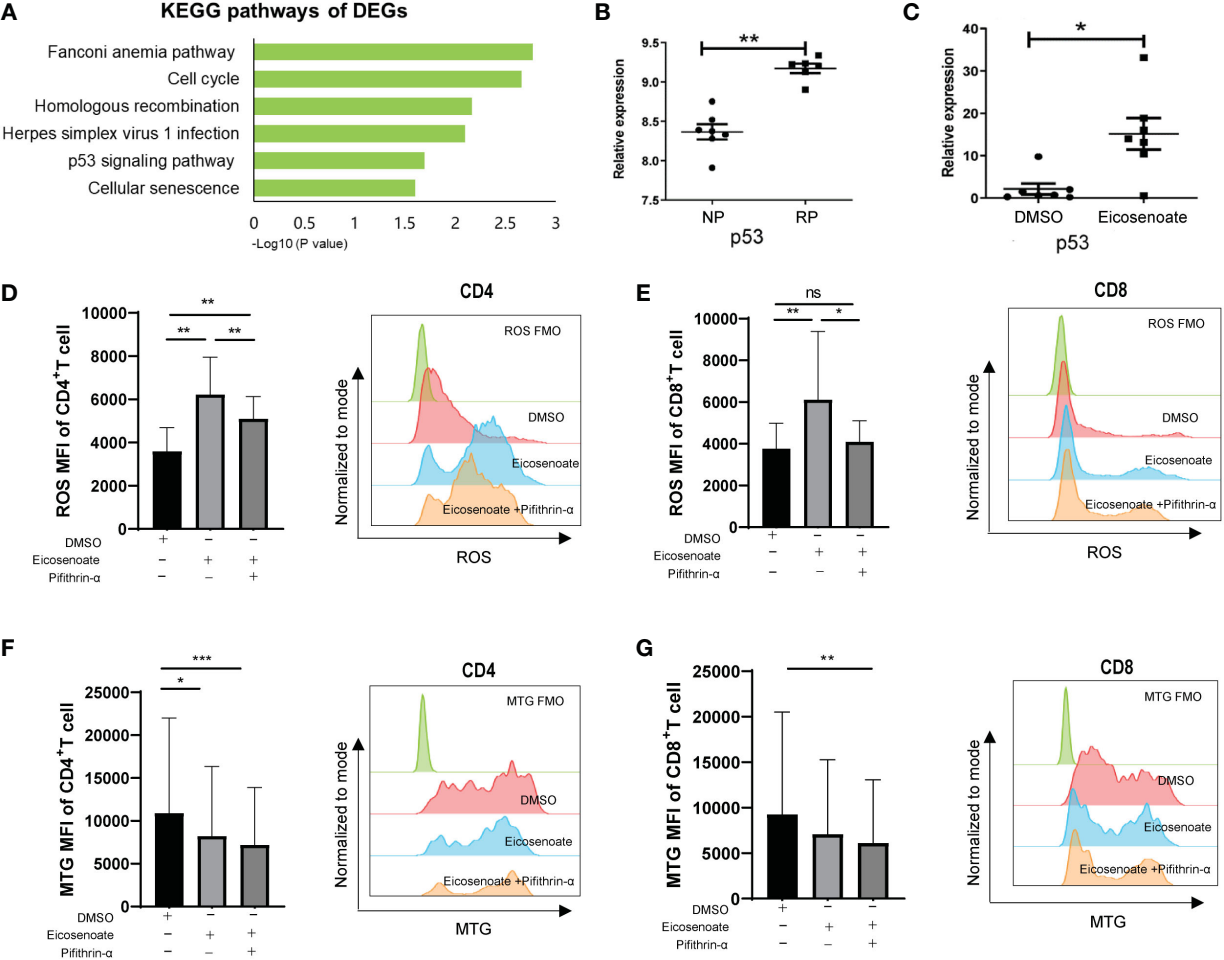
The p53 pathway participates in eicosenoate-induced impairment of T-cell mitochondrion function. **(A)** The top six signaling pathways enriched in 2,317 differentially expressed genes (DEGs) obtained from the analysis of previous transcriptome data (GSE44216) from rapid progressors (RPs) and normal progressors (NPs; adjusted *P* < 0.05). **(B)** p53 mRNA expression in PBMCs (GSE44216) was significantly increased in RPs (n = 6) compared with NPs (n = 7) (*P* < 0.01). **(C)** p53 mRNA expression in CD3^+^ T cells was significantly increased after co-incubation with 500μM eicosenoate and anti-CD3/CD28-coated Dynabeads (4:1 ratio) for 48 h *in vitro* (n = 7) (*P* < 0.05). p53 expression was quantified and normalized to that of GAPDH and expressed using the relative quantification method (2^−ΔΔCt^). **(D)** Primary CD3^+^ T cells were stimulated with anti-CD3/CD28-coated Dynabeads (4:1 ratio) and 500μM eicosenoate with or without 20μM p53 inhibitor pifithrin-α and incubated for 24 h, and mitochondrial ROS was detected. The p53 inhibitor pifithrin-α partially reduced the increase in ROS induced by eicosenoate in CD4^+^ T cells (n = 8). **(E)** The p53 inhibitor pifithrin-α prevented the increase in ROS induced by eicosenoate in CD8^+^ T cells to control levels (n = 8). **(F)** The eicosenoate-induced reduction in mitochondrial mass in CD4^+^ T cells was not restored by treatment with the p53 inhibitor pifithrin-α (n = 11). **(G)** The eicosenoate-induced reduction in mitochondrial mass in CD8^+^ T cells was not restored by treatment with the p53 inhibitor pifithrin-α (n = 11). Representative flow cytograms are shown in each panel. *P < 0.05, **P < 0.01, and ***P < 0.001.* P* values were calculated by Mann-Whitney test **(B, C)**, One-way ANOVA **(D)**, Friedman test **(E–G)**. ns, no significance.

To further explore the mechanism by which eicosenoate impairs mitochondrial function in T cells, we analyzed transcriptomic data in RP and NP PBMCs (GSE44216). KEGG pathway enrichment analysis was performed on the differentially expressed genes (DEGs) obtained by comparing RPs with NPs through GEO2R online analysis. The results showed that the DEGs were mainly enriched in six KEGG pathways (**Figure 4A**), among which the p53 signaling pathway was related to the regulation of mitochondrial function (36). We then studied whether the lipid metabolite eicosenoate impaired T-cell mitochondrial function by inducing p53 gene expression. We found that p53 mRNA expression was significantly increased in RPs compared with NPs (**Figure 4B**). Next, we found that eicosenoate could induce p53 mRNA expression in CD3+ T cells from HIV infected patients in vitro, according to qRT-PCR (**Figure 4C**). Then, we assessed changes in mitochondrial ROS and mitochondrial mass in CD4+ and CD8+ T cells based on anti-CD3/CD28 stimulation with or without the addition of a p53 pathway inhibitor pifithrin-α (20μM). The addition of pifithrin-α significantly reduced mitochondrial ROS in eicosenoate-treated CD4+ and CD8+ T cells but failed to increase mitochondrial mass (**Figures 4D–G**). These data suggest that the p53 pathway participated in the increase of mitochondrial ROS in CD4+ and CD8+ T cells induced by eicosenoate in HIV infection.

### 3.5 Mitochondrial antioxidant restores eicosenoate-induced T-cell dysfunction

**Figure 5:**
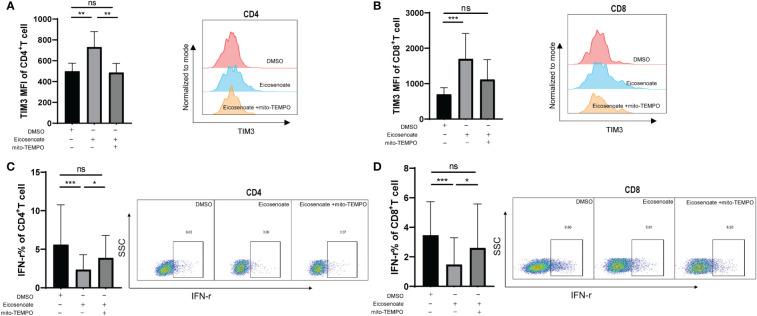
The mitochondrial antioxidant mito-TEMPO restores eicosenoate-induced T-cell dysfunction. **(A)** Primary CD3^+^ T cells were stimulated with 500μM eicosenoate and anti-CD3/CD28-coated Dynabeads (4:1 ratio) for 24 h, with or without 200μM mito-TEMPO, and the expression of the exhaustion indicator TIM3 on the T-cell surface was detected. Eicosenoate-induced increases in TIM3 MFI on CD4^+^ T cells was completely restored by treatment with 200μM mito-TEMPO (n = 6). **(B)** After treatment with 200μM mito-TEMPO, TIM3 MFI on CD8^+^T cells induced by eicosenoate tended to decrease (n = 6). **(C)** Primary CD3^+^ T cells were stimulated with 500μM eicosenoate and anti-CD3/CD28-coated Dynabeads (4:1 ratio) for 24 h, and IFN-γ expression was detected. The eicosenoate-induced reduction in IFN-γ in CD4^+^ T cells was restored by 200μM mito-TEMPO (n = 9). **(D)** The eicosenoate-induced reduction in IFN-γ in CD8^+^ T cells was prevented by treatment with 200μM mito-TEMPO (n = 9). Representative flow cytograms are shown in each panel. *P < 0.05, **P < 0.01, and ***P < 0.001.* P* values were calculated by Friedman test **(A–D)**. ns, no significance.

We demonstrated that eicosenoate induces mitochondrial ROS accumulation by inducing p53 expression. We next examined whether preventing increases in mitochondrial ROS levels could attenuate the inhibition of T-cell function induced by eicosenoate. Primary CD4+ and CD8+ T cells were treated with 500μM eicosenoate with or without the mitochondria-targeted antioxidant mito-TEMPO (200μM) for 24 h and expression of exhausted inhibitor TIM3 and the secretory function of T cells were determined (37). The results showed that the expression of TIM3 on the surface of CD4+ and CD8+ T cells stimulated by anti-CD3/CD28 for 24 h was significantly increased with treatment of the lipid metabolite eicosenoate. Subsequently, the expression of TIM3 on CD4+ T cells was significantly decreased with treatment of mito-TEMPO (**Figure 5A**). The expression of TIM3 on CD8+ T cells tended to decrease with treatment of mito-TEMPO, but was not statistical significance (**Figure 5B**). The IFN-γ expression was significantly decreased in CD4+ and CD8+ T cells induced by anti-CD3/CD28 in the presence of 500μM eicosenoate. Subsequently, IFN-γ expression in CD4+ and CD8+ T cells was significantly increased after treatment with mito-TEMPO for 24 h (**Figures 5C, D**). Mito-TEMPO could also significantly reduce the expression of PD-1, CD107a and mitochondrial ROS in eicosenoate treated CD3+ T cells (**Supplemental Figure 2**), which is consistent with previous studies that antioxidant could rescue the T cell dysfunction. These data suggest that mitochondrial ROS may be a target to rescue the impairment of T-cell function induced by the lipid metabolite eicosenoate.

## 4 Discussion

Metabolic pathways and metabolites instruct effector functions, differentiation, and gene expression of the immune system and might act as a potential target for the treatment of human diseases (38, 39). In this study, we found that HIV rapid disease progression could be predicted by an 11-lipid metabolite signature. Eicosenoate, one of the lipid metabolites upregulated in RPs, accelerated T-cell dysfunction by impairing mitochondrial function via induction of p53 expression. Treatment with a mitochondrial-targeting antioxidant decreased TIM3 expression and increased IFN-γ expression in eicosenoate-induced T cells.

Metabolomics can be used to map the perturbations of early biochemical changes in diseases, including cardiovascular diseases, neurologic diseases, diabetes mellitus, and infectious diseases (40). Studies have shown that metabolomic profiles can be used to differentiate between treatment-naïve chronic HIV-infected patients, anti-retroviral therapy (ART) patients, and NCs from one another (41–45); however, the impact of metabolites on disease progression is less understood. Few studies have identified the metabolites, such as alanine and glucose, that correlate with CD4+ T cells or viral load in HIV treatment-naïve patients (46). Recently, Scarpelini et al. found that five metabolites could be used to distinguish five RPs from five ART-immunologic non-responders (17). Zhang et al. showed that PG (O-18:0/18:0) could distinguish the plasma of nine RPs before seroconversion from that of 10 non-RPs before seroconversion (15). In the present study, an 11-lipid metabolite signature was identified that could predict rapid disease progression at ~120 days of infection with an accuracy of 91.9%, as measured by AUC. Rasheed, S and co-workers showed that HIV replication alone induces release of cellular enzymes and proteins that are significantly associated with biologically relevant processes involved in the synthesis, transport, and metabolism of lipids (47). Biofluid data demonstrated that alterations in lipid metabolites were seen in HIV patients receiving ART (41, 42, 44, 48, 49), which was linked to biomarkers of inflammation. The current data extend the importance of lipid metabolites by indicating that lipid metabolism plays an important role in rapid disease progression, and that lipid biomarkers can aid in the early differentiation between RPs and NPs.

Omics studies have provided important information for understanding the pathogenesis of disease (50, 51). Although deregulated lipid metabolism has been considered in HIV infection, the effect of lipid metabolites on immune T-cell function in HIV infection has not yet been determined. Here, we found that the long-chain fatty acid eicosenoate was upregulated in RPs and significantly suppressed T-cell proliferation and secretory function. Previous studies have shown that lipid metabolites negatively impact immune functions. Asarat, M and colleagues found that short-chain fatty acids upregulate production of anti-inflammatory cytokines in PBMCs, resulting in induction of CD4+CD25+ T-regulatory cells (52). Hara, Y and co-workers showed that long-chain fatty acids dose-dependently inhibit stimulated IFN-γ production by intraepithelial lymphocytes (31). Capric acid has inhibitory effects on osteoclast development via suppression of NF-κB signaling (30). Our results here indicate that the competition for nutrients and metabolites between HIV and immune cells influenced T-cell function.

Our study found for the first time that the long-chain fatty acid eicosenoate regulated mitochondrial homeostasis via p53 expression and reduced IFN-γ expression in in vitro activated T cells. Evidence has shown that mitochondrial metabolism determines immune T-cell differentiation, survival, and function and is a potential target for treating cancer (32, 53, 54). Manzo, T et al. showed that fatty acid accumulation in the tumor microenvironment impairs immune CD8+ T-cell function by reducing mitochondrial mass, mitochondrial respiration, and mitochondrial integrity (22). Jin, R and colleagues demonstrated that the long-chain fatty acid, linoleic acid, induced mitochondrial ROS accumulation via FABP5 to impair the anti-tumor immune T-cell response (33). Cells can benefit from low levels of ROS-mediated signals, but excessive ROS levels causes damage to mitochondrial proteins, organelle membranes, and DNA, thus changing the functional status of cells (34, 35). Fisicaro, P and colleagues demonstrated that the mitochondrial-targeting antioxidants mitoQ and mito-TEMPO restore anti-viral activity in exhausted HBV-specific T cells in chronic hepatitis B (55). Antioxidants that target mitochondria have also been shown to have therapeutic effects in Parkinson’s disease, metabolic syndrome, multiple sclerosis, sepsis (56–59). In chronic HIV infection, targeting mitochondrial dysfunction could restore the function of exhausted CD8+ T cells (23). Our in vitro experiments demonstrated for the first time that the mitochondria-targeting antioxidant mito-TEMPO intervenes immune T-cell TIM3 and IFN-γ expression caused by the lipid metabolite eicosenoate, showing that antioxidants may be effective drugs used to improve the function of immune T cells in HIV clinical treatment.

Overall, our study demonstrated that rapid HIV disease progression could be predicted by the profile of 11 lipid metabolites. The lipid metabolite eicosenoate impaired the immune function of T cells by inducing p53 expression and mitochondrial ROS accumulation. Our results provide an opportunity to understand the pathogenesis of HIV and clarify the immunometabolic alterations that occur during the progression of HIV disease.

## Data availability statement

The Metabolomic profiling data presented in this article are not readily available because of local policies. Requests to access the data should be directed to HS, E-mail: hongshang100@hotmail.com; Z-NZ, E-mail: zi_ning101@hotmail.com.

## Ethics statement

The studies involving human participants were reviewed and approved by Medical Science Research Ethics Committee of the First Hospital of China Medical University. The patients/participants provided their written informed consent to participate in this study.

## Author contributions 

HS and Z-NZ conceived and designed the experiments. S-YL, L-BY, H-BD, and ML performed the experiments and analyzed the data. J-NL, J-QL, JW, TT, Y-JF, and Y-JJ contributed reagents, materials, and analysis tools. HS, Z-NZ, S-YL, L-BY, H-BD, and ML wrote the article. All authors contributed to the article and approved the submitted version.

## Supplementary material

Supplementary Figure 1 Effect of the lipid metabolite Eicosenoate on markers of early and late T-cell activation. To investigate the effect of the lipid metabolite eicosenoate on markers of early and late T-cell activation, CD3^+^ T cells were stimulated with anti-CD3/CD28-coated Dynabeads (4:1 ratio) and 500 μM eicosenoate simultaneously. Primary CD3^+^ T cells were co-incubated with DMSO or eicosenoate plus anti-CD3/CD28-coated Dynabeads (4:1 ratio) for 24 h. The percentage of the expression of CD69, HLA-DR and CD25 on CD4^+^ and CD8^+^ T cells after eicosenoate treatment was shown **(A–F)**. *P < 0.05, **P < 0.01 and ***P < 0.001. P values were calculated by Paired t test **(A–F)**.

Supplementary Figure 2 Effects of the antioxidant mito-Tempo on mitochondrial reactive oxygen species (ROS), exhaustion marker PD-1 and functional marker CD107a in eicosenoate treated T cells. CD3^+^ T cells were stimulated with anti-CD3/CD28-coated Dynabeads (4:1 ratio) and 500 μM lipid metabolite eicosenoate simultaneously. **(A)** The expression of mitochondrial ROS MFI on the surface of CD3^+^ T cells was significantly decreased after a 24 h incubation with 200μM mito-Tempo (n=3). **(B)** The expression of PD-1 percent on the surface of CD3^+^ T cells was significantly decreased after a 24 h incubation with 200μM mito-Tempo (n=7). **(C)** The expression of CD107a MFI on the surface of CD8^+^ T cells was significantly decreased after a 24 h incubation with 200μM mito-Tempo (n=8). *P < 0.05. P values were calculated by Paired t test.
