# Supplementary material for: Altered lipid metabolites accelerate early dysfunction of T cells in HIV-infected rapid progressors by impairing mitochondrial function
Source: Front Immunol. 2023 Feb 17;14:1106881. doi: 10.3389/fimmu.2023.1106881 (PMC9981933; doi:10.3389/fimmu.2023.1106881)
Supplement: Supplementary file 1 [file Table_1.doc]

| **Supplemental Table 1.** Comparison of the expression of metabolites in early HIV infected patients and HIV negative controls | | | | |
| --- | --- | --- | --- | --- |
| **Super Pathway** | **Sub Pathway** | **Biochemical Name** | **Fold Change** | ***p*-value** |
| Peptide | Polypeptide | HWESASLLR | 37.87 | <0.0001 |
| Lipid | Phospholipid Metabolism | glycerophosphorylcholine (GPC) | 5.98 | <0.0001 |
| Peptide | Fibrinogen Cleavage Peptide | ADSGEGDFXAEGGGVR* | 44.59 | <0.0001 |
| Lipid | Sphingolipid Metabolism | sphinganine | 6.02 | <0.0001 |
| Lipid | Eicosanoid | 12-HETE | 17.08 | <0.0001 |
| Lipid | Sphingolipid Metabolism | sphingosine | 7.55 | <0.0001 |
| Lipid | Lysolipid | 1-(1-enyl-oleoyl)-GPE (P-18:1)* | 3.73 | <0.0001 |
| Peptide | Polypeptide | HWESASXX* | 13.20 | <0.0001 |
| Peptide | Polypeptide | XHWESASXXR* | 342.09 | <0.0001 |
| Amino Acid | Glutathione Metabolism | 5-oxoproline | 1.92 | <0.0001 |
| Amino Acid | Glutamate Metabolism | glutamate | 2.37 | <0.0001 |
| Carbohydrate | Glycogen Metabolism | maltose | 3.94 | <0.0001 |
| Lipid | Lysolipid | 1-(1-enyl-palmitoyl)-GPE (P-16:0)* | 3.16 | <0.0001 |
| Peptide | Polypeptide | bradykinin, hydroxy-pro(3) | 29.00 | <0.0001 |
| Lipid | Polyunsaturated Fatty Acid (n3 and n6) | arachidonate (20:4n6) | 2.73 | <0.0001 |
| Energy | TCA Cycle | malate | 1.95 | <0.0001 |
| Lipid | Glycerolipid Metabolism | glycerol 3-phosphate | 2.21 | <0.0001 |
| Lipid | Lysolipid | 1-palmitoyl-GPA (16:0) | 1.65 | <0.0001 |
| Lipid | Lysolipid | 1-stearoyl-GPS (18:0)* | 3.77 | <0.0001 |
| Amino Acid | Glutathione Metabolism | glutathione, oxidized (GSSG) | 0.31 | <0.0001 |
| Amino Acid | Methionine, Cysteine, SAM and Taurine Metabolism | cysteine | 0.58 | <0.0001 |
| Xenobiotics | Food Component/Plant | stachydrine | 3.96 | <0.0001 |
| Peptide | Polypeptide | bradykinin | 20.65 | <0.0001 |
| Lipid | Eicosanoid | 15-HETE | 12.06 | <0.0001 |
| Lipid | Fatty Acid, Monohydroxy | 2-hydroxystearate | 1.45 | <0.0001 |
| Lipid | Lysolipid | 1-stearoyl-GPE (18:0) | 1.44 | <0.0001 |
| Lipid | Monoacylglycerol | 1-oleoylglycerol (18:1) | 2.63 | 0.0001 |
| Lipid | Fatty Acid, Monohydroxy | 2-hydroxypalmitate | 1.30 | 0.0001 |
| Lipid | Lysolipid | 1-(1-enyl-stearoyl)-GPE (P-18:0)* | 3.26 | 0.0001 |
| Energy | TCA Cycle | alpha-ketoglutarate | 2.84 | 0.0001 |
| Cofactors and Vitamins | Hemoglobin and Porphyrin Metabolism | bilirubin (Z,Z) | 0.49 | 0.0001 |
| Nucleotide | Purine Metabolism, Adenine containing | adenosine 5'-monophosphate (AMP) | 2.38 | 0.0002 |
| Peptide | Polypeptide | HXGXA* | 6.30 | 0.0002 |
| Lipid | Polyunsaturated Fatty Acid (n3 and n6) | dihomo-linolenate (20:3n3 or n6) | 1.90 | 0.0002 |
| Carbohydrate | Aminosugar Metabolism | erythronate* | 1.28 | 0.0004 |
| Energy | TCA Cycle | succinate | 1.54 | 0.0004 |
| Peptide | Gamma-glutamyl Amino Acid | gamma-glutamylmethionine | 0.65 | 0.0005 |
| Amino Acid | Tryptophan Metabolism | kynurenine | 1.28 | 0.0005 |
| Amino Acid | Glutamate Metabolism | glutamine | 0.71 | 0.0007 |
| Peptide | Dipeptide Derivative | N-acetylcarnosine | 0.77 | 0.0007 |
| Nucleotide | Pyrimidine Metabolism, Uracil containing | pseudouridine | 1.11 | 0.0007 |
| Peptide | Polypeptide | bradykinin, des-arg(9) | 2.24 | 0.0009 |
| Peptide | Gamma-glutamyl Amino Acid | gamma-glutamylvaline | 1.22 | 0.0010 |
| Energy | TCA Cycle | succinylcarnitine | 0.77 | 0.0011 |
| Lipid | Eicosanoid | 5-HETE | 12.11 | 0.0017 |
| Amino Acid | Leucine, Isoleucine and Valine Metabolism | isovalerylcarnitine | 0.68 | 0.0019 |
| Amino Acid | Phenylalanine and Tyrosine Metabolism | 4-hydroxyphenylacetate | 0.67 | 0.0022 |
| Lipid | Long Chain Fatty Acid | arachidate (20:0) | 1.36 | 0.0026 |
| Lipid | Lysolipid | 1-palmitoyl-GPE (16:0) | 1.34 | 0.0029 |
| Lipid | Polyunsaturated Fatty Acid (n3 and n6) | eicosapentaenoate (EPA; 20:5n3) | 1.78 | 0.0037 |
| Lipid | Lysolipid | 1-stearoyl-GPI (18:0) | 1.65 | 0.0046 |
| Lipid | Fatty Acid, Monohydroxy | 3-hydroxydecanoate | 1.54 | 0.0050 |
| Lipid | Inositol Metabolism | chiro-inositol | 3.73 | 0.0051 |
| Amino Acid | Creatine Metabolism | creatinine | 0.90 | 0.0053 |
| Amino Acid | Urea cycle; Arginine and Proline Metabolism | dimethylarginine (SDMA + ADMA) | 1.21 | 0.0054 |
| Peptide | Gamma-glutamyl Amino Acid | gamma-glutamylisoleucine* | 1.28 | 0.0060 |
| Lipid | Long Chain Fatty Acid | nonadecanoate (19:0) | 1.28 | 0.0061 |
| Lipid | Polyunsaturated Fatty Acid (n3 and n6) | docosadienoate (22:2n6) | 1.43 | 0.0061 |
| Nucleotide | Pyrimidine Metabolism, Uracil containing | uridine | 0.84 | 0.0063 |
| Carbohydrate | Pentose Metabolism | glycerate | 1.25 | 0.0065 |
| Amino Acid | Urea cycle; Arginine and Proline Metabolism | N-methylproline | 2.22 | 0.0069 |
| Amino Acid | Tryptophan Metabolism | N-acetyltryptophan | 0.81 | 0.0072 |
| Lipid | Fatty Acid, Monohydroxy | 13-HODE + 9-HODE | 3.32 | 0.0078 |
| Lipid | Lysolipid | 2-stearoyl-GPC (18:0)* | 1.40 | 0.0089 |
| Lipid | Steroid | cortisol | 1.28 | 0.0108 |
| Nucleotide | Purine Metabolism, Guanine containing | N2,N2-dimethylguanosine | 1.27 | 0.0116 |
| Lipid | Polyunsaturated Fatty Acid (n3 and n6) | docosahexaenoate (DHA; 22:6n3) | 1.36 | 0.0119 |
| Carbohydrate | Glycolysis, Gluconeogenesis, and Pyruvate Metabolism | 1,5-anhydroglucitol (1,5-AG) | 1.18 | 0.0121 |
| Lipid | Polyunsaturated Fatty Acid (n3 and n6) | adrenate (22:4n6) | 1.32 | 0.0131 |
| Lipid | Polyunsaturated Fatty Acid (n3 and n6) | docosapentaenoate (n6 DPA; 22:5n6) | 1.41 | 0.0165 |
| Lipid | Polyunsaturated Fatty Acid (n3 and n6) | docosapentaenoate (n3 DPA; 22:5n3) | 1.59 | 0.0165 |
| Amino Acid | Alanine and Aspartate Metabolism | aspartate | 1.22 | 0.0165 |
| Amino Acid | Leucine, Isoleucine and Valine Metabolism | N-acetylleucine | 0.86 | 0.0171 |
| Lipid | Secondary Bile Acid Metabolism | taurocholenate sulfate* | 0.65 | 0.0189 |
| Amino Acid | Methionine, Cysteine, SAM and Taurine Metabolism | N-acetylmethionine | 0.82 | 0.0192 |
| Nucleotide | Purine Metabolism, (Hypo)Xanthine/Inosine containing | urate | 0.90 | 0.0214 |
| Lipid | Lysolipid | 1-palmitoyl-GPI (16:0)* | 1.28 | 0.0227 |
| Amino Acid | Methionine, Cysteine, SAM and Taurine Metabolism | methionine | 0.88 | 0.0246 |
| Amino Acid | Tryptophan Metabolism | serotonin | 0.81 | 0.0255 |
| Lipid | Fatty Acid Metabolism(Acyl Carnitine) | hydroxybutyrylcarnitine* | 1.34 | 0.0263 |
| Carbohydrate | Pentose Metabolism | pyruvate | 0.86 | 0.0266 |
| Amino Acid | Phenylalanine and Tyrosine Metabolism | N-acetyltyrosine | 0.83 | 0.0281 |
| Amino Acid | Phenylalanine and Tyrosine Metabolism | 4-hydroxyphenylpyruvate | 0.79 | 0.0295 |
| Lipid | Polyunsaturated Fatty Acid (n3 and n6) | dihomo-linoleate (20:2n6) | 1.43 | 0.0325 |
| Lipid | Lysolipid | 2-palmitoyl-GPC (16:0)* | 1.20 | 0.0331 |
| Cofactors and Vitamins | Nicotinate and Nicotinamide Metabolism | trigonelline (N'-methylnicotinate) | 0.78 | 0.0338 |
| Lipid | Long Chain Fatty Acid | stearate (18:0) | 1.14 | 0.0364 |
| Amino Acid | Leucine, Isoleucine and Valine Metabolism | 2-methylbutyrylcarnitine (C5) | 0.84 | 0.0372 |
| Nucleotide | Purine Metabolism, Adenine containing | N1-methyladenosine | 0.93 | 0.0379 |
| Lipid | Sphingolipid Metabolism | sphingosine 1-phosphate | 0.72 | 0.0451 |
| Xenobiotics | Benzoate Metabolism | 4-vinylphenol sulfate | 0.66 | 0.0460 |
|  |  |  |  |  |
