# Supplementary material for: Altered lipid metabolites accelerate early dysfunction of T cells in HIV-infected rapid progressors by impairing mitochondrial function
Source: Front Immunol. 2023 Feb 17;14:1106881. doi: 10.3389/fimmu.2023.1106881 (PMC9981933; doi:10.3389/fimmu.2023.1106881)
Supplement: Supplementary file 2 [file Table_2.doc]

| **Supplemental Table 2.** Comparison of metabolites in RPs and NPs | |  |  |  |
| --- | --- | --- | --- | --- |
| **Biochemical Name** | **Super Pathway** | **Sub Pathway** | **Fold change RP/NP** | ***p*-value** |
| tetradecanedioate | Lipid | Fatty Acid, Dicarboxylate | **2.13** | 0.0130 |
| caprate (10:0) | Lipid | Medium Chain Fatty Acid | **2.04** | 0.0015 |
| myristoleate (14:1n5) | Lipid | Long Chain Fatty Acid | **2.00** | 0.0397 |
| caprylate (8:0) | Lipid | Medium Chain Fatty Acid | **1.69** | 0.0015 |
| 12,13-DiHOME | Lipid | Fatty Acid, Dihydroxy | **1.64** | 0.0232 |
| 10-nonadecenoate (19:1n9) | Lipid | Long Chain Fatty Acid | **1.61** | 0.0153 |
| 9,10-DiHOME | Lipid | Fatty Acid, Dihydroxy | **1.59** | 0.0326 |
| 3-hydroxydecanoate | Lipid | Fatty Acid, Monohydroxy | **1.54** | 0.0359 |
| eicosenoate (20:1n9 or 11) | Lipid | Long Chain Fatty Acid | **1.54** | 0.0406 |
| laurate (12:0) | Lipid | Medium Chain Fatty Acid | **1.49** | 0.0143 |
| myristate (14:0) | Lipid | Long Chain Fatty Acid | **1.39** | 0.0141 |
| 7-alpha-hydroxy-3-oxo-4-cholestenoate (7-Hoca) | Lipid | Sterol | **1.33** | 0.0411 |
| nonadecanoate (19:0) | Lipid | Long Chain Fatty Acid | **1.27** | 0.0209 |
| kynurenine | Amino Acid | Tryptophan Metabolism | **1.19** | 0.0360 |
| mannose | Carbohydrate | Fructose, Mannose and Galactose Metabolism | **1.19** | 0.0420 |
| glucose | Carbohydrate | Glycolysis, Gluconeogenesis, and Pyruvate Metabolism | **1.18** | 0.0116 |
| dimethylarginine (SDMA + ADMA) | Amino Acid | Urea cycle; Arginine and Proline Metabolism | **1.16** | 0.0320 |
| urate | Nucleotide | Purine Metabolism, (Hypo)Xanthine/Inosine containing | **0.90** | 0.0252 |
| gamma-glutamylleucine | Peptide | Gamma-glutamyl Amino Acid | **0.88** | 0.0466 |
| 1-linoleoyl-GPC (18:2) | Lipid | Lysolipid | **0.88** | 0.0492 |
| propionylcarnitine | Lipid | Fatty Acid Metabolism (also BCAA Metabolism) | **0.80** | 0.0106 |
| 5alpha-pregnan-3beta,20alpha-diol disulfate | Lipid | Steroid | **0.79** | 0.0170 |
| 21-hydroxypregnenolone disulfate | Lipid | Steroid | **0.78** | 0.0313 |
| pregnen-diol disulfate* | Lipid | Steroid | **0.72** | 0.0128 |
| xylonate | Carbohydrate | Pentose Metabolism | **0.72** | 0.0124 |
| 4-hydroxyphenylpyruvate | Amino Acid | Phenylalanine and Tyrosine Metabolism | **0.70** | 0.0304 |
| 4-hydroxyphenylacetate | Amino Acid | Phenylalanine and Tyrosine Metabolism | **0.68** | 0.0230 |
| 1-linolenoyl-GPC (18:3)* | Lipid | Lysolipid | **0.67** | 0.0467 |
| gamma-CEHC | Cofactors and Vitamins | Tocopherol Metabolism | **0.65** | 0.0223 |
| 3-phenylpropionate (hydrocinnamate) | Amino Acid | Phenylalanine and Tyrosine Metabolism | **0.61** | 0.0333 |
| hippurate | Xenobiotics | Benzoate Metabolism | **0.53** | 0.0022 |
| deoxycholate | Lipid | Secondary Bile Acid Metabolism | **0.38** | 0.0369 |
| cholate | Lipid | Primary Bile Acid Metabolism | **0.19** | 0.0041 |
|  |  |  |  |  |
|  |  |  |  |  |
