# Supplementary figures and images for: Altered lipid metabolites accelerate early dysfunction of T cells in HIV-infected rapid progressors by impairing mitochondrial function
Source: Front Immunol. 2023 Feb 17;14:1106881. doi: 10.3389/fimmu.2023.1106881 (PMC9981933; doi:10.3389/fimmu.2023.1106881)

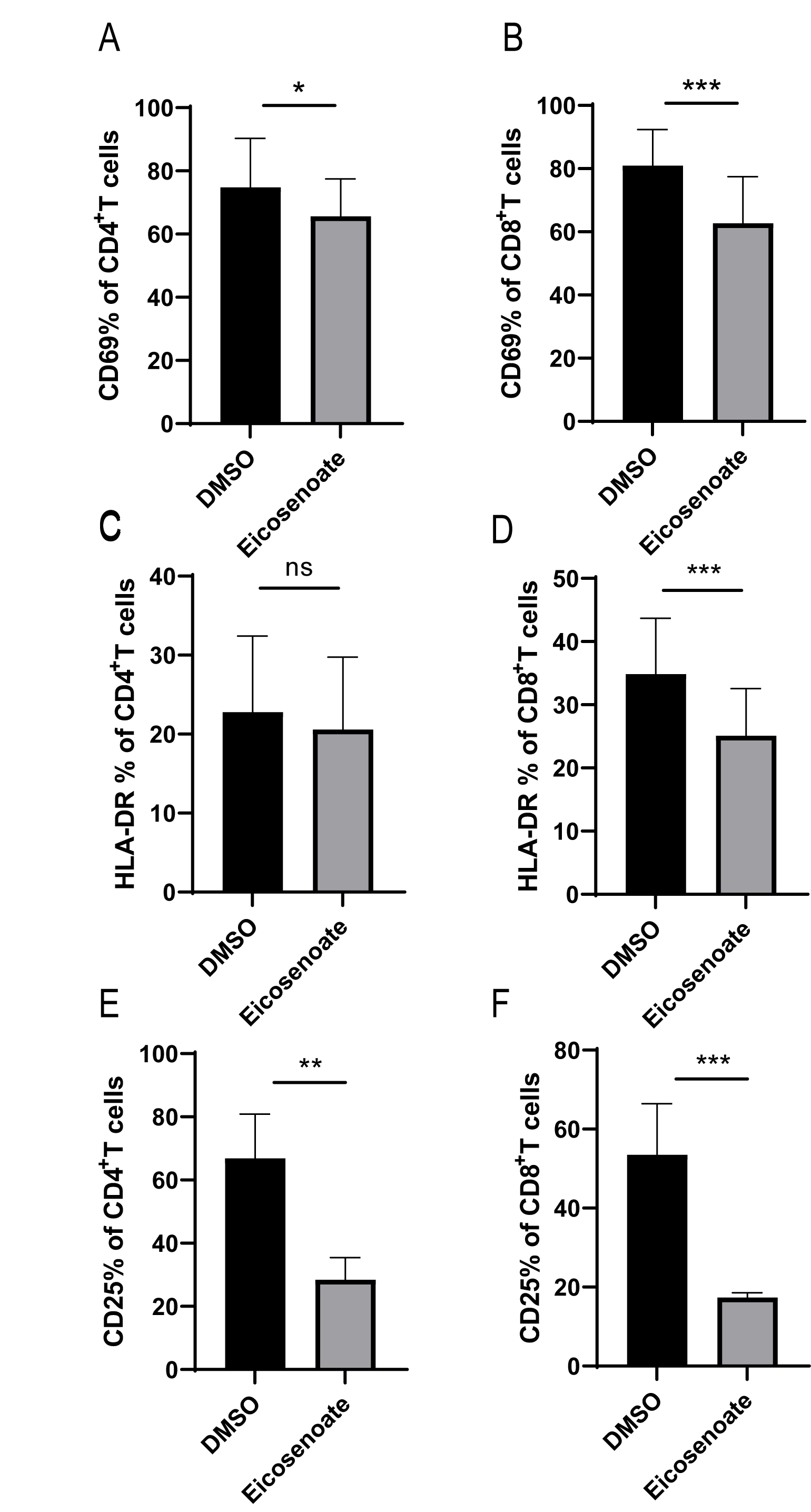

Supplement: Supplementary Figure 1 — Effect of the lipid metabolite Eicosenoate on markers of early and late T-cell activation. To investigate the effect of the lipid metabolite eicosenoate on markers of early and late T-cell activation, CD3+ T cells were stimulated with anti-CD3/CD28-coated Dynabeads (4:1 ratio) and 500 μM eicosenoate simultaneously. Primary CD3+ T cells were co-incubated with DMSO or eicosenoate plus anti-CD3/CD28-coated Dynabeads (4:1 ratio) for 24 h. The percentage of the expression of CD69, HLA-DR and CD25 on CD4+ and CD8+ T cells after eicosenoate treatment was shown (A–F). *P < 0.05, **P < 0.01 and ***P < 0.001. P values were calculated by Paired t test (A–F). [file Image_1.tif]

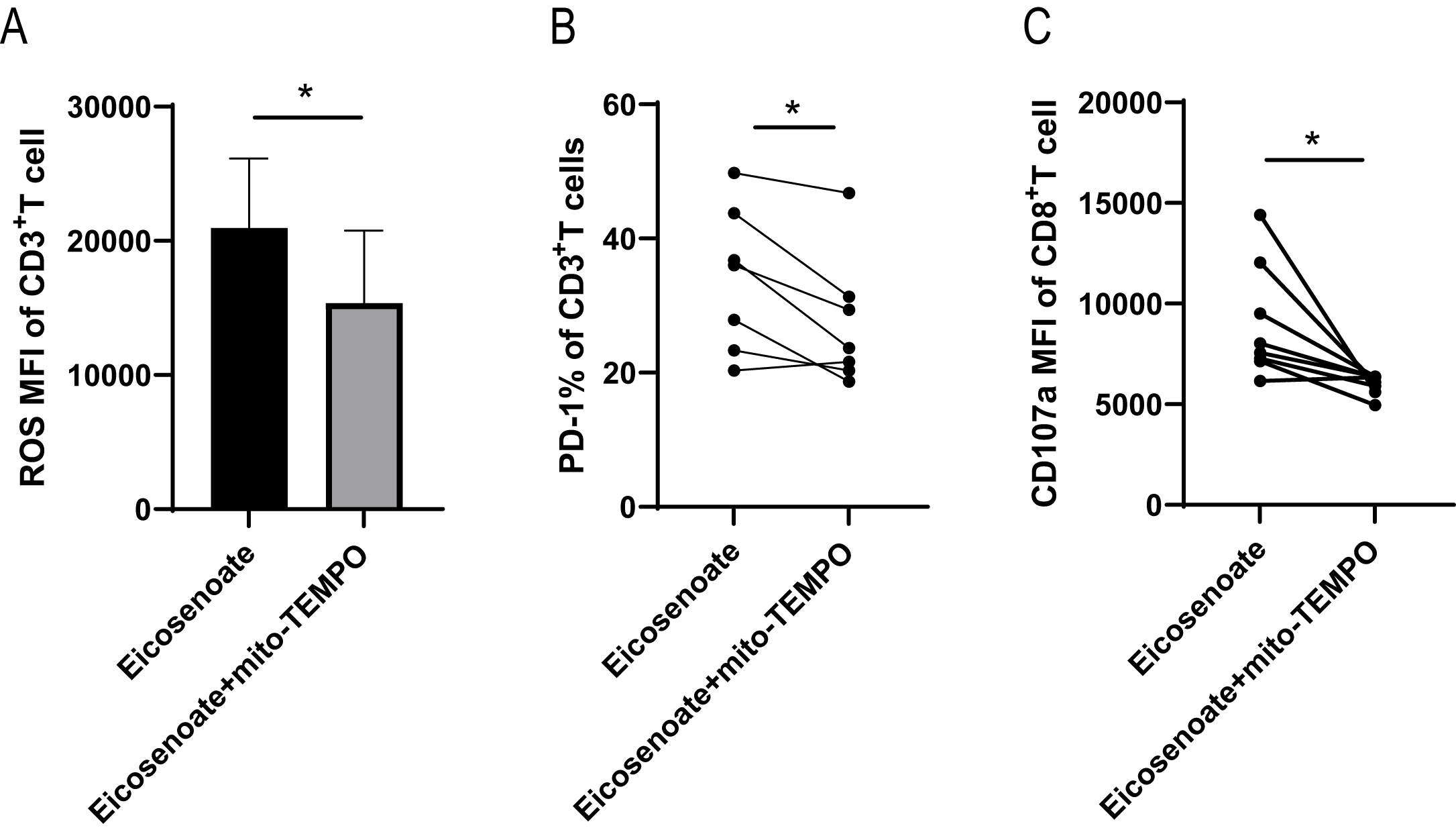

Supplement: Supplementary Figure 2 — Effects of the antioxidant mito-Tempo on mitochondrial reactive oxygen species (ROS), exhaustion marker PD-1 and functional marker CD107a in eicosenoate treated T cells. CD3+ T cells were stimulated with anti-CD3/CD28-coated Dynabeads (4:1 ratio) and 500 μM lipid metabolite eicosenoate simultaneously. (A) The expression of mitochondrial ROS MFI on the surface of CD3+ T cells was significantly decreased after a 24 h incubation with 200μM mito-Tempo (n=3). (B) The expression of PD-1 percent on the surface of CD3+ T cells was significantly decreased after a 24 h incubation with 200μM mito-Tempo (n=7). (C) The expression of CD107a MFI on the surface of CD8+ T cells was significantly decreased after a 24 h incubation with 200μM mito-Tempo (n=8). *P < 0.05. P values were calculated by Paired t test. [file Image_2.tif]
